# Supplementary material for: Spatial oxidation of L-plastin downmodulates actin-based functions of tumor cells
Source: Nat Commun. 2019 Sep 9;10:4073. doi: 10.1038/s41467-019-11909-z (PMC6733871; doi:10.1038/s41467-019-11909-z)
Supplement: Supplementary file 1 — Supplementary Information [file 41467_2019_11909_MOESM1_ESM.pdf]

Spatial oxidation of L-plastin downmodulates actin based functions of tumor cells

Balta et al.

Supplementary Figures

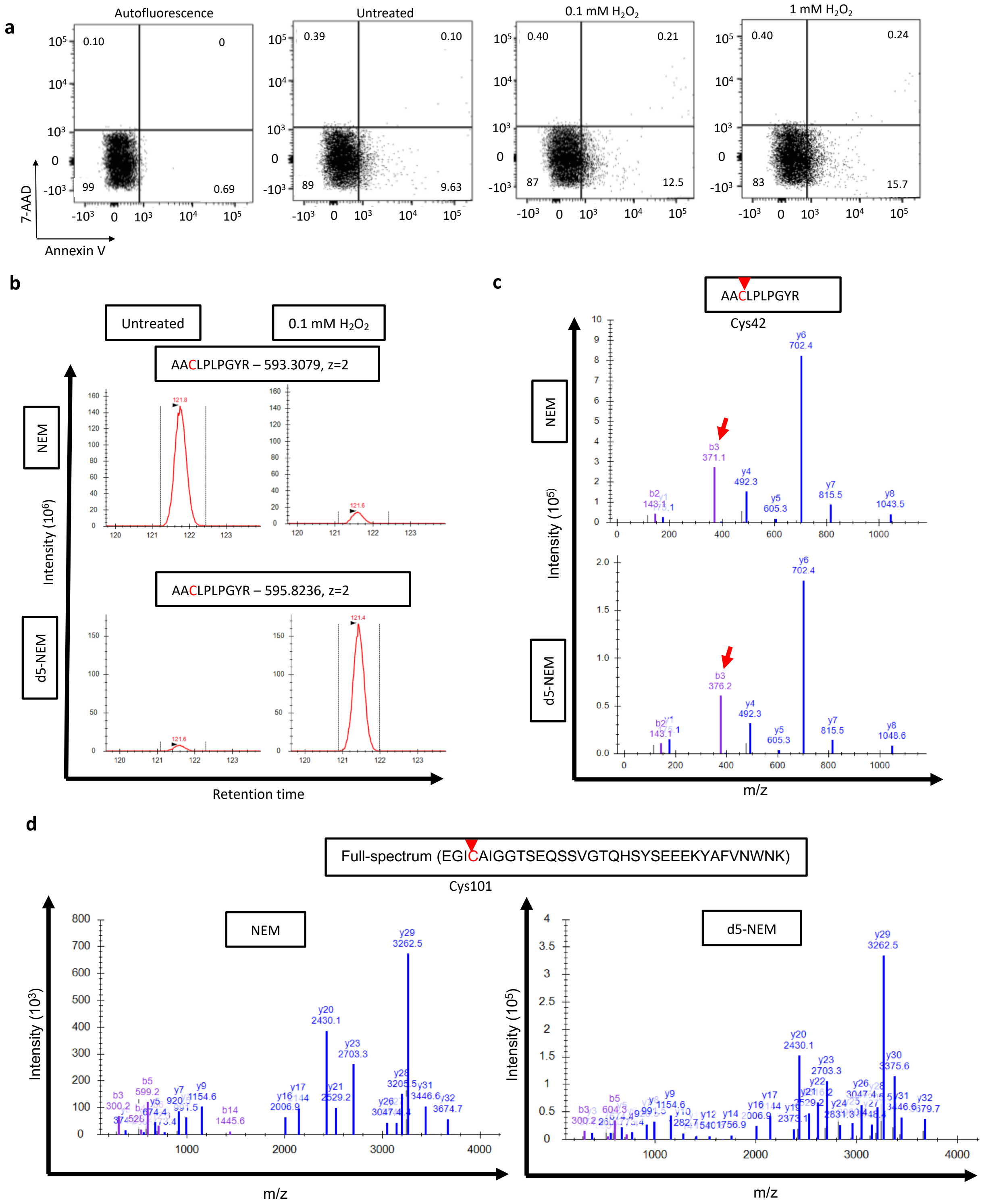

Supplementary Figure 1. Analysis of oxidation on Cys42 and Cys101 at MS1 and MS2 levels. **a** Assessment of cell viability upon H_2O_2 treatment. PBTs were treated with H_2O_2 for 30 min with the indicated concentrations. Cells were then stained for Annexin V and 7-AAD. Shown are representative flow cytometry dot plots for Annexin V and 7-AAD (n = 3). H_2O_2 treatment of the cells and following differential alkylation reactions were performed as described (see Fig. 1). **b** Representative extracted-ion chromatograms (EICs) showing the retention time versus intensity for the LPL peptides aa 39-48 labeled with NEM (upper graphs) and d5-NEM (lower graphs) in the absence (left) and presence (right) of 0.1 mM H_2O_2. **c** MS2 spectra of both the NEM- and d5-NEM-labeled peptides (aa 39-48). The red arrow indicates the distinctive ion (b3 ion) that carries the respective NEM label type. **d** Representative MS2 spectra at full m/z range of the NEM-labelled (left) and the d5-NEM-labelled (right) aa 97–131 peptide (n = 3).

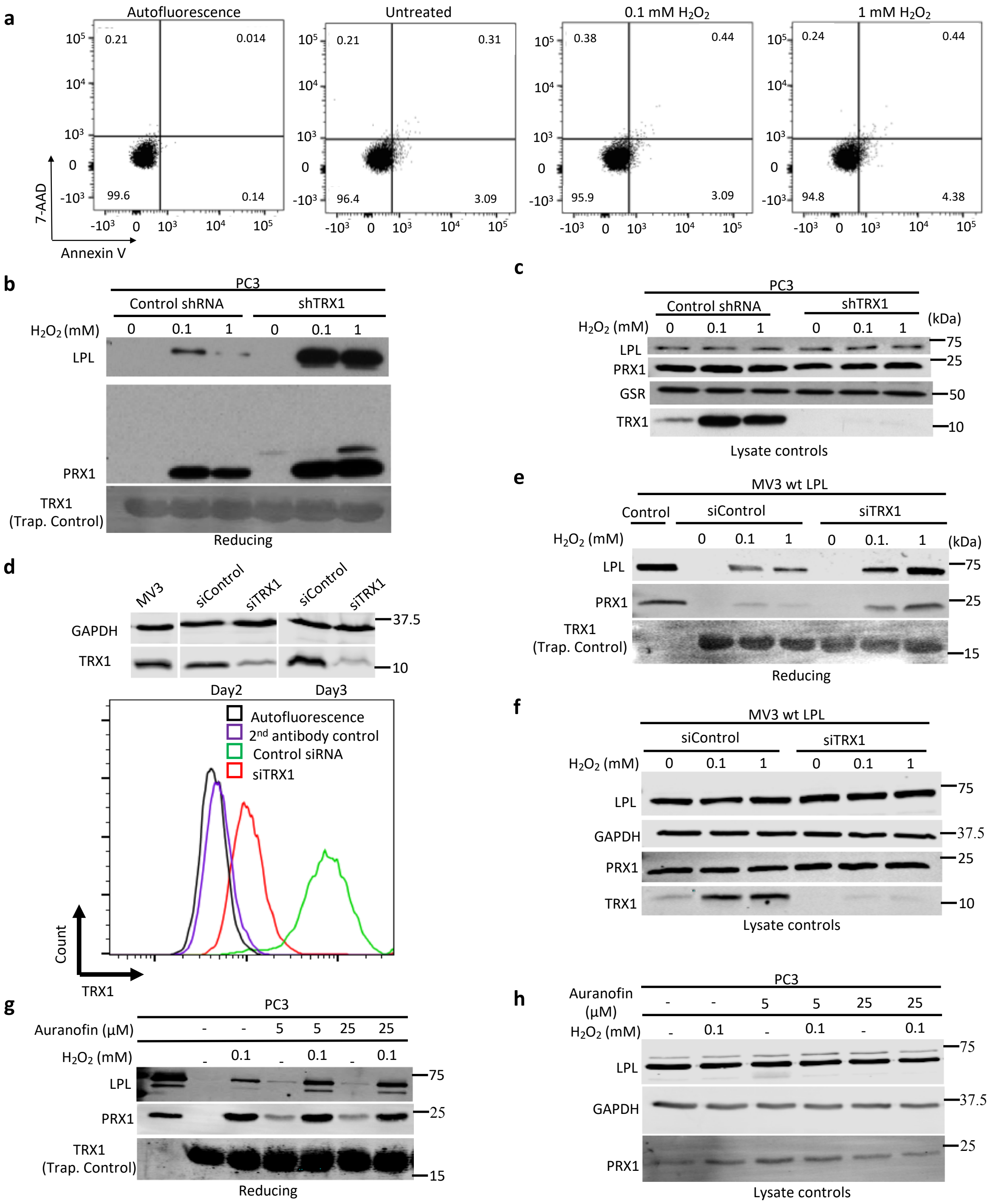

Supplementary Figure 2. Prooxidative challenge of cells lacking the TRX1 system. **a** Assessment of HEK293 cell viability upon H_2O_2 treatment. HEK293 cells were treated with H_2O_2 for 5 min with the indicated concentrations. Thereafter, cells were stained for Annexin V and 7-AAD. Shown are representative flow cytometry dot plots for Annexin V and 7-AAD ($n \geq 3$). **b** Representative immunoblots showing the TRX1 trapping of LPL and PRX1 in control and TRX1 knockdown PC3 cells. Samples were run under reducing conditions (n = 3). **c** Corresponding lysate controls of the trapping reactions in PC3 cells (n = 4). **d** TRX1 knockdown in MV3 cells by siRNAs**.** The MV3 cells stably expressing LPL constructs were transfected with siRNAs. TRX1 expression was monitored at protein level by western blotting and flow cytometry. Shown are representative immunoblots at day 2 and day 3. Representative flow cytometry histogram shows the TRX1 level three days post siRNA transfection (n = 3). **e** Representative reducing gel-immunoblots and IP controls showing TRX1 trapping of LPL and PRX1 in control and TRX1 knockdown MV3 cells (n = 3). **f** Corresponding lysate controls of the trapping reactions in MV3 cells (n = 3). **g** Representative reducing gel-immunoblots and IP controls showing TRX1 trapping of LPL and PRX1 in PC3 cells in the presence or absence of auranofin. TRX1 trapping (trap.) control represents the amount of TRX1 trapping mutant used in each of the samples (n = 3). **h** Corresponding lysate controls of the trapping reactions in PC3 cells (n = 3).

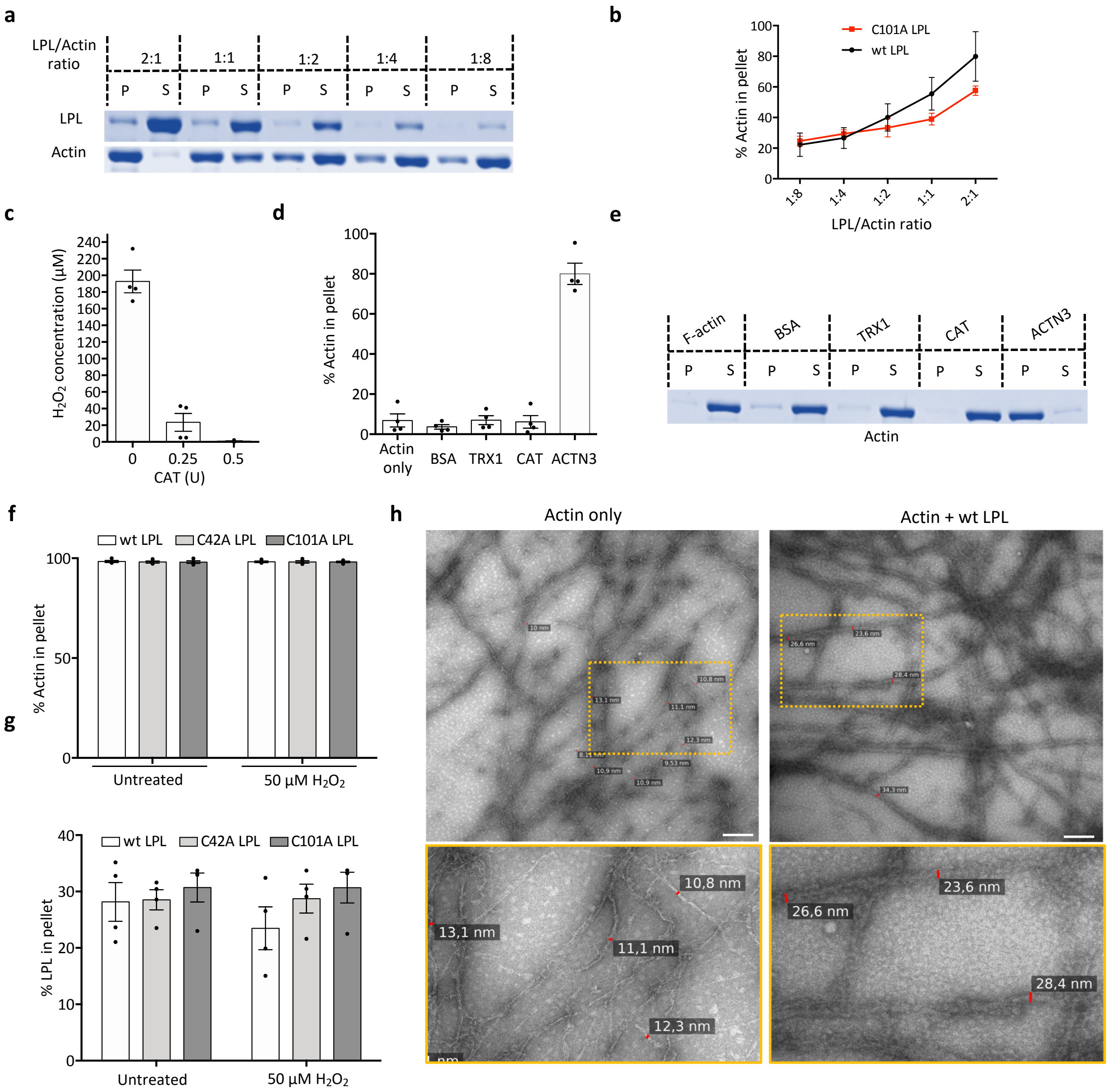

Supplementary Figure 3. Actin-binding and bundling capacity of LPL. **a** Representative Coomassie-stained gels showing bundling of recombinant wt LPL with various LPL/actin ratios (P: pellet, S: supernatant). Recombinant LPL was coincubated with G-actin in actin polymerization buffer for 4 h. The actin bundles were separated from filaments and G-actin by low-speed centrifugation. The pellet and supernatant fractions were loaded on SDS gel and immunoblotted for LPL and actin (n = 3). **b** Percent quantification of actin in the pellet for different LPL/actin ratios (n = 3). **c** Removal of H_2O_2 via CAT prior to actin bundling. 5 min prior to coincubation of LPL and G-actin, 0.25 or 0.5 U of CAT was added to H_2O_2 treated LPL. The remaining H_2O_2 was quantified using H_2O_2 detection kit (n = 4). **d** Percent quantification of actin in pellet. Actin-bundling assay was performed in the presence of the indicated proteins. BSA and alpha-actinin (ACTN3) represent negative and positive controls of actin-bundling, respectively. 3 µg/sample TRX1 and 0.5 U CAT mimic ROS treatment conditions. **e** Representative Coomassie-stained gels of actin-bundling controls (n = 4). **f-g** Percent quantification of **f** actin and **g** LPL in the pellet after actin co-sedimentation (n = 4). Recombinant LPL was coincubated with G-actin in actin polymerization buffer as described above. Thereafter, unbound LPL and G-actin were separated from LPL-bound F-actin and actin bundles by a high-speed centrifugation. The pellet and supernatant fractions were loaded on SDS gel and immunoblotted for LPL and actin (n = 4). Percent protein in the pellet was calculated by taking the ratio of the corresponding signal in the pellet fraction over total intensity (supernatant and pellet). The data are presented as the mean ± SEM. **h** Electron micrographs of actin filaments (upper panel) and actin bundles (lower panel). Following F-actin polymerization and bundling, samples were negatively stained and imaged by TEM. Scale bar = 200 nm.

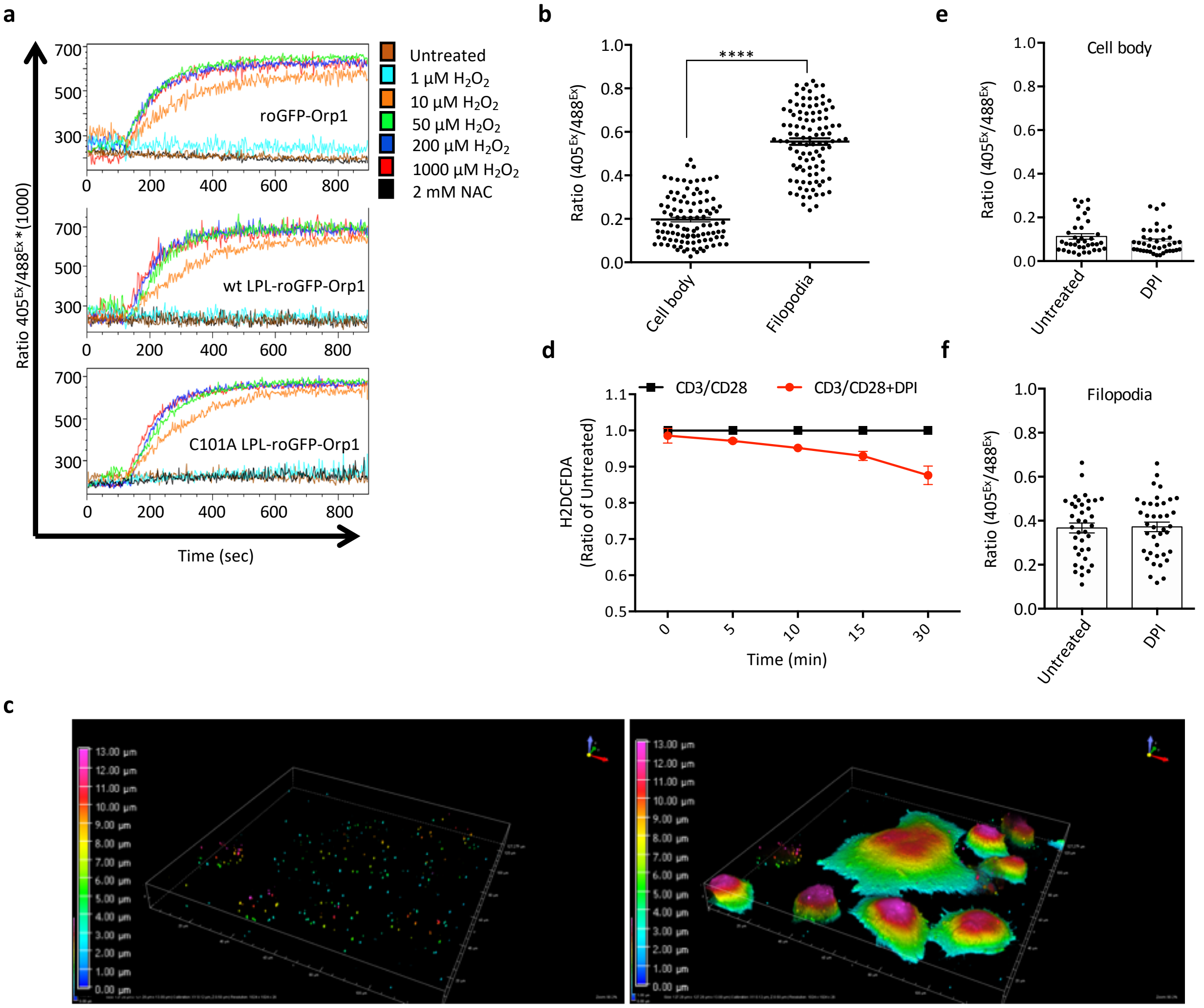

Supplementary Figure 4. Analysis of spatial oxidation of LPL. **a** Representative response curve of the roGFP-Orp1 (upper panel), wt LPL (middle panel) and C101A LPL (lower panel) fused to roGFP-Orp1 in MV3 cells under prooxidative (H_2O_2) or antioxidative (NAC) conditions. Ratio imaging was performed using LSR II (n = 3). **b** Quantification of the ratio of oxidized and reduced ($405^{Ex}/488^{Ex}$) wt LPL roGFP-Orp1 in the cell body and filopodia of MV3 cells. Ratio imaged cells were analyzed using NIS elements. Three different filopodia extensions and three areas throughout the cell body were selected for each cell. Ratio of ox/red roGFP in each selected area was taken for analysis. 103 cells were analyzed in three different experiments. **c** In-depth projection of z-stacks showing the PLA puncta (left) or LPL eGFP signal (right) at different z-positions. MV3 wt LPL eGFP cells were imaged using confocal microscopy upon PLA staining. Color shows the depth in z-position of cells (n ≥ 3). **d** DPI treatment of activated PBTs. PBTs were stained with 5 µM CM-H2DCFDA sensor for 15 min and activated with plate bound anti-CD3/anti-CD28 antibodies for the indicated periods in the presence or absence of 10 µM DPI. Thereafter, the mean fluorescent intensity of the sensor was measured by flow cytometry. Data were created by normalization of MFI of the sensor in DPI treated cells to that of untreated cells for each time point (n = 3). **e-f** Quantification of roGFP-Orp1 ratiometric measurements ($405^{Ex}/488^{Ex}$) in MV3 **e** cell body and **f** in filopodia after DPI treatment. 35 cells were imaged and analyzed in at least three different experiments as described. *P* values were calculated by t-test (b). The data are presented as the mean ± SEM (n = 3; **** = $p < 0.0001$).

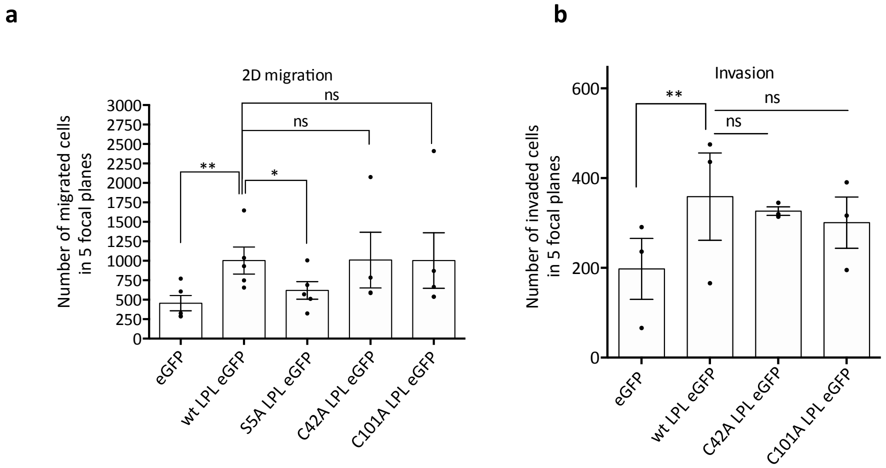

Supplementary Figure 5. LPL enhances migration and invasion of MV3 cells. **a** Quantification of migration of MV3 cells expressing the indicated LPL eGFP constructs on 2D transwell chambers and **b** on 3D invasion chambers. Upon migration or invasion, cells at the lower compartment of the transwell inserts were mounted on coverslips. At least five xy focal planes were imaged per sample using LSM. *P* values were calculated by one-way ANOVA. The data are presented as the mean ± SEM ($n \geq 3$; * = $p < 0.05$, ** = $p < 0.01$, ns = nonsignificant).

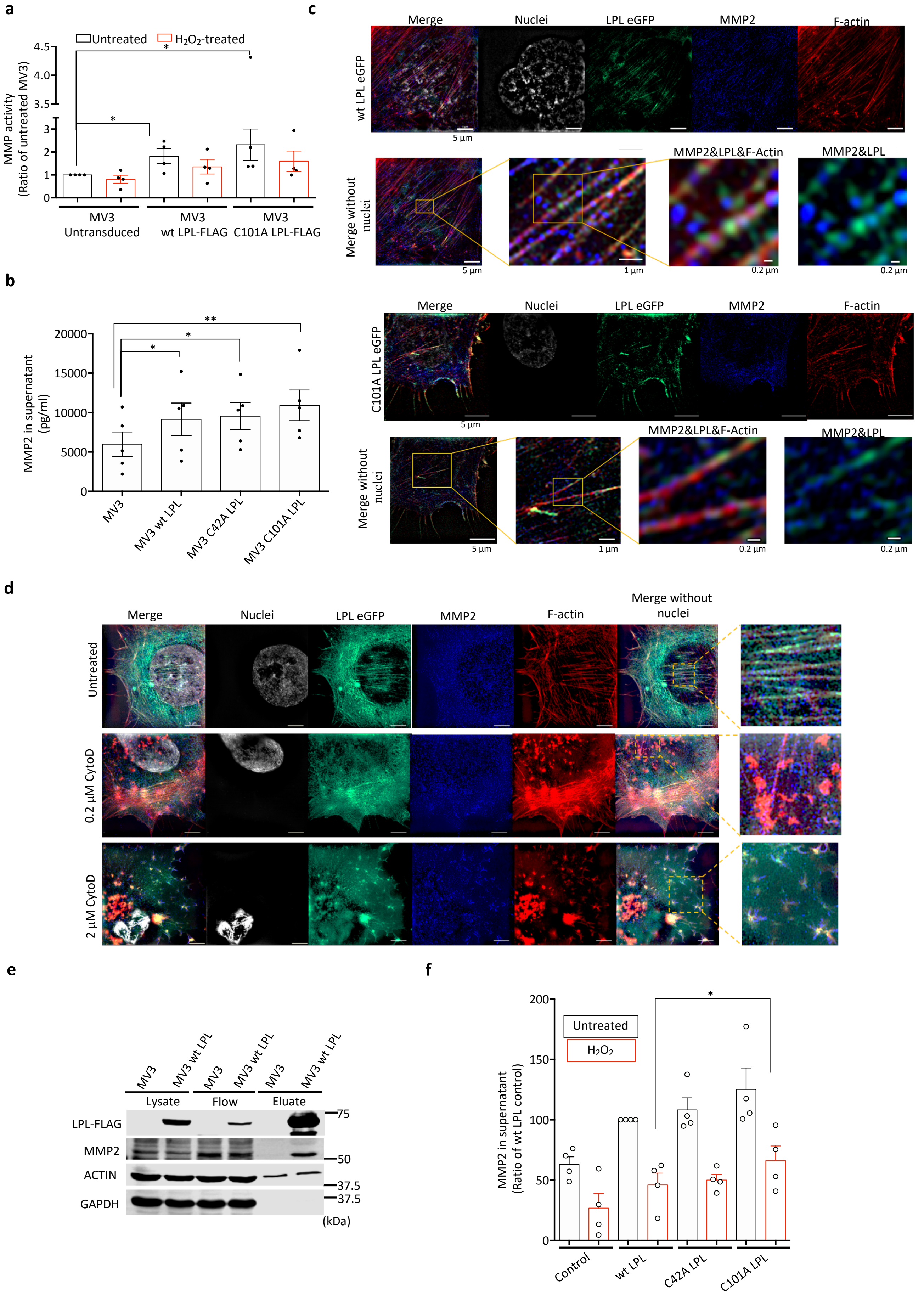
a
Untreated
H2O2-treated
MMP activity
(Ratio of untreated MV3)
4.5
4.0
3.5
3
2
1
0
*
*
MV3
Untransduced
MV3
wt LPL-FLAG
MV3
C101A LPL-FLAG
b
MMP2 in supernatant
(pg/ml)
20000
15000
10000
5000
0
**
*
*
MV3
MV3 wt LPL
MV3 C42A LPL
MV3 C101A LPL
c
Merge
Nuclei
LPL eGFP
MMP2
F-actin
wt LPL eGFP
5 µm
Merge without nuclei
MMP2&LPL&F-Actin
MMP2&LPL
1 µm
0.2 µm
C101A LPL eGFP
d
Merge
Nuclei
LPL eGFP
MMP2
F-actin
Merge without nuclei
Untreated
0.2 µM CytoD
2 µM CytoD
e
MV3
MV3 wt LPL
Lysate
Flow
Eluate
LPL-FLAG
MMP2
ACTIN
GAPDH
75
50
37.5
37.5
(kDa)
f
MMP2 in supernatant
(Ratio of wt LPL control)
200
150
100
50
0
*
Untreated
H2O2
Control
wt LPL
C42A LPL
C101A LPL

Supplementary Figure 6. Correlation between LPL and MMP2. **a** MMP activity in the supernatant of LPL negative MV3 cells (untransduced) and MV3 cells expressing wt LPL or C101A LPL in the absence (black bars) or presence (red bars) of 25 µM H_2O_2. MV3 cells were seeded onto the gelatin-coated wells and the supernatant was collected 3 h post H_2O_2 treatment. Relative MMP activity was calculated by normalization to control (untreated untransduced MV3 cells) (n = 4). **b** Concentration of MMP2 in the supernatant of MV3 cells or MV3 cells expressing the indicated LPL constructs. Supernatants of MV3 cells were collected and MMP2-specific ELISA was performed (n = 4). **c** SIM imaging of MV3 wt LPL cells (upper panels) and MV3 C101A LPL cells (lower panels) on gelatin-coated coverslips. Cells were allowed to adhere on gelatin-coated coverslips for 3 h. Cells were then fixed and imaged using an N-SIM microscopy. Shown are nuclei (white), LPL eGFP (green), MMP2 (blue) and F-actin (red). The merged images represent the digital overlay of all four colors or as specified. Lower graphs show magnification of the selected areas (n ≥ 3). Scale bars are given underneath each image; 100 x magnification **d** Dissociation of actin stress fibers via Cytochalasin D (Cyto D) treatment leads to disappearance of MMP2-fiber pattern in the cells. Representative 3D SIM images of MV3 wt LPL cells upon Cyto D treatment with the indicated concentrations. Magnified merge images show colocalization of LPL, MMP2 on F-actin stress fibers (untreated). 0.2 µM and 2 µM Cyto D treatment groups represent loss of F-actin stress fibers and resulting loss of MMP2 or LPL signature on fibers (n = 3). **e** FLAG-IP in control or LPL-FLAG expressing cells. Cell lysates were coincubated with FLAG beads for 120 min. After removal of the unbound fraction, FLAG antibody bound proteins were affinity-purified using FLAG peptides. Eluates were run on SDS-polyarcrylamide gel and immunoblotted for the indicated proteins (n=3). **f** Fold change in MMP2 concentration in the supernatant of untreated (black bars) and H_2O_2 treated (red bars) LPL negative MV3 cells or MV3 cells expressing the indicated LPL constructs relative to control (untreated MV3 wt LPL). *P* values were calculated by one-way ANOVA (b) and t-test (a,f). The data are presented as the mean ± SEM (n = 4; * = $p < 0.05$).

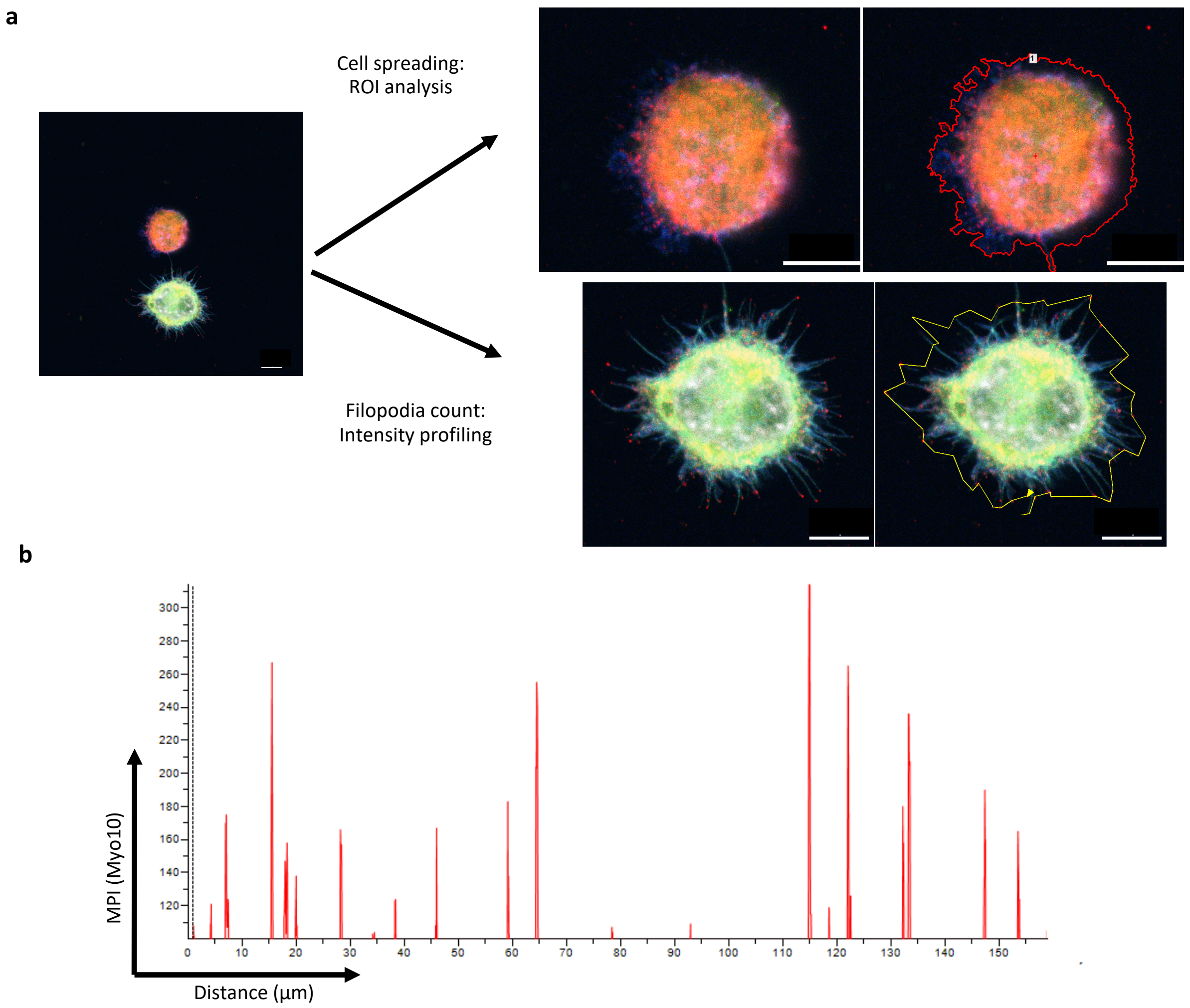

Supplementary Figure 7. Analysis of filopodia counts in PC3 cells. **a** Representative merge image of PC3 cells and analysis of region of interest (ROI) for assessment of cell spreading and analysis of filopodia counting. The intensity profile is drawn at the tip of each filopodia around the cell. **b** Mean pixel intensity (MPI) graph is automatically created based on the intensity profile. A threshold is set above background as 2-fold. In the representative experiment, it was set as 100. After thresholding, each peak in the histogram was counted as one filopodium (n ≥ 3). Scale bar = 10 µm; 100 x magnification.

Supplementary Tables

Cysteine Position	Peptide sequence	d5-NEM peptides MS1-level identification	d5-NEM peptides MS2-level identification	Sulfonylation (SO_3H) MS2-level identification
42	AACLPLPGYR	3	3	3
101	EGICAIGGTSEQSSVGTQHSYSEEEK	3	3	-
101	EGICAIGGTSEQSSVGTQHSYSEEEKYAFVNWINK	3	3	3
140	ALENDPDCR	2	1	2
164	HVIPMNPNTNDLFNAVGDGIVLCK	1	-	1
206	LTPFTIQENLNLALNSASAIGCHVVNIGAEDLK	1	-	-
283	WANYHLENAGCNK	-	-	1
336	EKDDIQRAECMLQQAER	1	-	1
336	AECMLQQAER	3	2	1
346	LGCRQFVTATDVVR	-	-	1
460	KLENCNYAVELGK	2	-	2
460	LENCNYAVELGK	1	-	-
618	MVMTVFACLMGK	1	-	-

Supplementary Table 1. Low levels of oxidation on cysteines of LPL. Summary of LC-MS/MS results showing identification of oxidized LPL cysteines. PBTs were treated with 0.1 mM and 1 mM H_2O_2 for 30 min. Cells were then lysed and differentially alkylated using NEM, DTT and d5-NEM as described before. Shown are the MS1- and MS2-levels of detected peptides carrying a d5-NEM or MS2-identification of sulfonyl-modification. The numbers indicate how often the respective peptide was identified out of the three replicate experiments (n = 3).

Primer name	Restriction-enzyme	Sequence	Generated construct
pLJM1 LPL eGFP-F	NheI	GACTGCTAGCATGGCCAGAGGATCAGTGTC	pLJM1 LPL eGFP
pLJM1 LPL eGFP-R	AgeI	GACTACCGGTCGCACCCTCTTCATTCCTTTCC	
pLJM1_LPL FLAG-R	BstBI	GACTTTCGAATTACTTGTCATCGTCGTCCTTGTAATC	pLJM1 LPL-FLAG
pLJM1_LPL FLAG-F	AfeI	GACTAGCGCTATGGCCAGAGGATCAGTGTC	
pGEX2T-LPL-F	BAMHI	ACACGGATCCATGGCCAGAGGATCAGTGTC	pGEX2T- GST-LPL
pGEX2T-LPL-R	ECORI	GATCGAATTCTCACACCCTCTTCATTCCTT	
roGFP-Orp1-F	AgeI	GATCACCGGTAATGGTGAGCAAGGGCGA	pLJM1 LPL-roGFP-Orp1
roGFP-Orp1-R	BstBI	GATCTTCGAACTATTCCACCTCTTTCAAAAG	

Supplementary Table 2. Primers used for cloning of LPL sequences into lentiviral vectors. For all the cloning procedures, pEGFPN1-LPL was used as the backbone. The primer pairs (forward (F) and reverse (R)) and the target vector name is given under primer name. The restriction enzyme at the 5`- end of the primer sequence is used for restriction-ligation based cloning of the corresponding construct. For amplification of roGFP-Orp1, pLCPX-roGFP-Orp1 was used as backbone.

Supplementary Note 1

Detailed explanation of Supplementary Figure 6.

Degradation of extracellular matrix is achieved via the release and activity of MMPs. Therefore, it was tempting to speculate that MMP activity or release could be directly or indirectly regulated by LPL and its oxidation. To address this point, first the total MMP activity was assessed in supernatants of cells seeded on gelatin-coated plates. A marked increase in MMP activity was detected in the supernatants of MV3 cells expressing wt LPL but a correlation between redox regulation of LPL and MMP activity was not observed (Supplementary Fig. 6a). MMP activity in the supernatants is affected by many factors including ROS. Therefore, we analyzed the release of MMPs by measuring the presence of different MMPs in the supernatants. Among others, only MMP2 was significantly higher in the supernatants of LPL expressing cells (Supplementary Fig. 6b).

To further delineate the relationship between LPL and the increased release of MMP2, we took advantage of super-resolution microscopy (SIM). Wt or C101A LPL eGFP expressing MV3 cells were first allowed to adhere on gelatin-coated coverslips. Thereafter, cells were fixed and stained for MMP2 and F-actin. SIM analysis showed that MMP2 is closely associated with LPL on the F-actin filaments (stress fibers) (Supplementary Fig. 6c-d). A magnified view of the images clearly showed a special signature of these three components (Supplementary Fig. 6c-d). Along the F-actin filaments, LPL and MMP2 showed both colocalization and a repeated frequency signal. LPL, MMP2 and F-actin also colocalized in invadopodial structures (data not shown). To substantiate these findings, FLAG-tagged LPL expressing MV3 cells or control cells were lysed, the FLAG-tagged LPL was pulled down and analyzed for coimmunoprecipitation of MMP2. Interestingly, MMP2 coimmunoprecipitated with LPL in LPL-FLAG expressing cells (Supplementary Fig. 6e). Collectively, these findings reveal a clear link between the matrix degradation capacity of cells and LPL expression.

Finally, we tested whether LPL thiol switches play a role in the release of MMP2. Therefore, an MMP2 specific ELISA was performed using the supernatants of MV3 cells expressing wt LPL, C42A LPL, or C101A LPL, that were seeded on gelatin matrix in the presence or absence of 25 µM H_2O_2. H_2O_2-dependent inhibition of MMP2 release occurred in all groups but slightly less in MV3 cells expressing the C101A LPL compared to wt LPL expressing cells (Supplementary Fig. 6f). Altogether, these findings showed that LPL enhanced the release of MMP2 to actively degrade the gelatin matrix. Moreover, the local release of MMP2 at invadopodia may be positively influenced by LPL, while oxidation of LPL does not strongly influence this release.
