# Supplementary material for: Spatial oxidation of L-plastin downmodulates actin-based functions of tumor cells
Source: Nat Commun. 2019 Sep 9;10:4073. doi: 10.1038/s41467-019-11909-z (PMC6733871; doi:10.1038/s41467-019-11909-z)
Supplement: Supplementary file 3 — Reporting Summary [file 41467_2019_11909_MOESM3_ESM.pdf]

nature research

Corresponding author(s): Prof. Dr. med. Yvonne Samstag
Institute of Immunology
Section Molecular Immunology
Heidelberg University
Im Neuenheimer Feld 305, 69120
Heidelberg/Germany
Email: yvonne.samstag@urz.uni-heidelberg.de

Last updated by author(s): 23/7/19

Reporting Summary

Nature Research wishes to improve the reproducibility of the work that we publish. This form provides structure for consistency and transparency in reporting. For further information on Nature Research policies, see Authors & Referees and the Editorial Policy Checklist.

Statistics

For all statistical analyses, confirm that the following items are present in the figure legend, table legend, main text, or Methods section.

n/a	Confirmed	
☐	☒	The exact sample size (n) for each experimental group/condition, given as a discrete number and unit of measurement
☐	☒	A statement on whether measurements were taken from distinct samples or whether the same sample was measured repeatedly
☐	☒	The statistical test(s) used AND whether they are one- or two-sided<br>*Only common tests should be described solely by name; describe more complex techniques in the Methods section.*
☒	☐	A description of all covariates tested
☒	☐	A description of any assumptions or corrections, such as tests of normality and adjustment for multiple comparisons
☐	☒	A full description of the statistical parameters including central tendency (e.g. means) or other basic estimates (e.g. regression coefficient) AND variation (e.g. standard deviation) or associated estimates of uncertainty (e.g. confidence intervals)
☐	☒	For null hypothesis testing, the test statistic (e.g. F, t, r) with confidence intervals, effect sizes, degrees of freedom and P value noted<br>*Give P values as exact values whenever suitable.*
☒	☐	For Bayesian analysis, information on the choice of priors and Markov chain Monte Carlo settings
☒	☐	For hierarchical and complex designs, identification of the appropriate level for tests and full reporting of outcomes
☒	☐	Estimates of effect sizes (e.g. Cohen's d, Pearson's r), indicating how they were calculated

Our web collection on statistics for biologists contains articles on many of the points above.

Software and code

Policy information about availability of computer code

Data collection

Program	Vendor
FACSDiva v. 6.1.2	BD Biosciences
Imagestudio V3	LI-COR Biosciences
NIS elements	Nikon
Jeol JEM1400	Jeol Ltd
Xcalibur	Thermo Fischer Scientific

Data analysis

Program	Vendor
Snapgene V3	Snapgene
Prism 6 V.6	Graphpad Software
Office programs	Microsoft
Maxquant V.8.1	Max Planck Institute of Biochemistry
Skyline	Open-source/skyline
Inkscape	Open-source/Inkscape
FlowJo v. 9.8	Tree star
Imagestudio V3	LI-COR Biosciences
EMMeasure	TVIPS
NIS elements	Nikon

For manuscripts utilizing custom algorithms or software that are central to the research but not yet described in published literature, software must be made available to editors/reviewers. We strongly encourage code deposition in a community repository (e.g. GitHub). See the Nature Research guidelines for submitting code & software for further information.

Data

Policy information about availability of data

All manuscripts must include a data availability statement. This statement should provide the following information, where applicable:

- Accession codes, unique identifiers, or web links for publicly available datasets
- A list of figures that have associated raw data
- A description of any restrictions on data availability

The authors declare that all data of this study are available within the article and the supplementary materials or from the corresponding author upon request. The source data underlying Figs. 1-9 and Supplementary Figs. 1-7 are provided as a single source data file.

Field-specific reporting

Please select the one below that is the best fit for your research. If you are not sure, read the appropriate sections before making your selection.

☒ Life sciences ☐ Behavioural & social sciences ☐ Ecological, evolutionary & environmental sciences

For a reference copy of the document with all sections, see nature.com/documents/nr-reporting-summary-flat.pdf

Life sciences study design

All studies must disclose on these points even when the disclosure is negative.

Sample size	No statistical methods were used to determine sample size.<br>Specifically, the biochemical experiments for identification of thiol switches on LPL were performed at least three times. The figure legends contain the corresponding information.<br>All of the in vitro experiments were performed at least three times. Before the hypothesis was tested, for each experimental setting, an establishment approach was followed. In particular, this gave us the chance to monitor the effect size of the positive response (positive controls) and the basal states. When a clear cut/reproducible setting was established, the influence of pro-oxidative settings on cellular functions and correlation to LPL oxidation was tested. For this, the experiments were performed at least three times.
Data exclusions	No data was excluded from this study.
Replication	The number of replicates for each figure presented in this study is clearly explained in figure legends and in the methods section. Each method used in this study was initially tested/established under control conditions with positive and negative controls. Only when a stable/ reproducible experimental setting was established, we proceeded to test our hypothesis. The particular reason for this step-by-step approach was to determine the variabilities of the experimental settings and to optimize them accordingly. When the effect range of treatments in relation to redox regulation are narrow, such a well-defined experimental strategy was a must for us to follow.
Randomization	Not applicable.
Blinding	Investigators were not blinded.

Reporting for specific materials, systems and methods

We require information from authors about some types of materials, experimental systems and methods used in many studies. Here, indicate whether each material, system or method listed is relevant to your study. If you are not sure if a list item applies to your research, read the appropriate section before selecting a response.

Materials & experimental systems

n/a	Involved in the study
☐	☒ Antibodies
☐	☒ Eukaryotic cell lines
☒	☐ Palaeontology
☒	☐ Animals and other organisms
☒	☐ Human research participants
☒	☐ Clinical data

Methods

n/a	Involved in the study
☒	☐ ChIP-seq
☐	☒ Flow cytometry
☒	☐ MRI-based neuroimaging

Antibodies

Antibodies used

Primary antibodies used in the study are listed below.

Antibody	Host/Clone	Usage	Vendor	Catalog number
Anti-ACTIN	Mouse /AC-74	WB	Sigma	A5316
Anti-CD3	Mouse/OKT3	IC	In-house	n.a.

Anti-CD28	Mouse/C3H	IC	BD Pharmingen	555725
Anti-DUOX1	Rb/H-9	WB, IF	Santacruz	sc-393096
Anti-DUOX2	Rb/polyclonal	WB, IF	Novusbio	NB110-61576
Anti-Cysteine Sulfenic Acid (dimedone)	Rb/poyclonal	WB, IF, FC	Merck	07-2139
Anti-GAPDH	Mouse/ 6C5	WB	Ambion	AM4300
Anti-Glutathione reductase (GSR)	Rabbit	WB	Abcam	ab16801
Anti-LPL	Mouse/LPL4A.1	WB, IF, FC	Thermo Fischer Scientific	MA5-11921
Anti-MMP2	Rb/poyclonal	WB, IF	NovusBio	NB200-193
Anti-MMP2	Mouse/M6303D01	WB, IF	Biolegend	679902
Anti-MYO10	Rb/polyclonal	IF	Sigma	22430002
Anti-NOX4	Rb/poyclonal	WB, IF	NovusBio	NB200-193
Anti-NOX4	Rb/polyclonal	WB, IF	Proteintech	14347-1-AP
Anti-PRX1	Rb/polyclonal	WB	Thermo Fischer Scientific	LF-PA0095
Anti-TRX1	Mouse/2G11	WB, IF, FC	BD Pharmingen	559969
Anti-TRXR1	Mouse/489804	WB, IF	R&D Systems	MAB7428

Secondary antibodies used in the study are listed below.

Antibody	Host/Clone	Usage	Vendor	Catalog number
anti-mouse IRDye-680RD	Goat	WB	LI-COR Biosciences	926-68072
anti-mouse IRDye-800CW	Donkey	WB	LI-COR Biosciences	926-32212
anti-rabbit IRDye-680RD	Donkey	WB	LI-COR Biosciences	926-68073
anti-rabbit-IRDye-800CW	Donkey	WB	LI-COR Biosciences	926-32213
anti-rabbit HRP	Goat	WB	Dianova	111-035-045
anti-Mouse HRP	Goat	WB	Dianova	111-035-045
anti-mouse Cy3	Donkey/polyclonal	IF	Dianova	711-165-152
anti-rabbit Cy3	Donkey/polyclonal	IF	Dianova	115-165-146
anti-mouse AF405	Goat	IF	Thermo Fischer Scientific	A-31553
anti-rabbit AF405	Goat	IF	Thermo Fischer Scientific	A-31556

Validation

The antibodies are used /purchased if/when they are validated for their respective use. For uncertainties regarding the specificity, a completely different antibody was tested for the respective usage.

Eukaryotic cell lines

Policy information about cell lines

Cell line source(s)

Human primary T-cells: Healthy volunteers. This study was approved by the Ethics Committee of Heidelberg University (S-269/2015).
MV3 (melanoma cell line): Provided by Dr. van Muijen, University Hhospital Nijmegen, Netherlands
PC3 (prostate cancer cell line): Provided by Dr. M. Cecchini, University of Bern, Switzerland
HEK293: ATCC
HEK293T: Provided by Dr. Steve Boulont, University Hospital Heidelberg

Authentication

The cell lines from ATCC are authenticated.
MV3 and PC3 cell lines were tested by genome sequencing (DSMZ, Braunschweig, Germany).

Mycoplasma contamination

All cell lines were tested negative for Mycoplasm contamination.

Commonly misidentified lines (See ICLAC register)

Not applicable

Flow Cytometry

Plots

Confirm that:

☒ The axis labels state the marker and fluorochrome used (e.g. CD4-FITC).

☒ The axis scales are clearly visible. Include numbers along axes only for bottom left plot of group (a 'group' is an analysis of identical markers).

☐ All plots are contour plots with outliers or pseudocolor plots.

☒ A numerical value for number of cells or percentage (with statistics) is provided.

Methodology

Sample preparation

Prior to staining, cells were washed once with PBS and stained for Annexin-FITC and 7-AAD according to the manufacturer's instructions. Samples were directly measured by multispectral flow cytometry. The power for lasers and PMT were set according to the Cytometer Setup & Tracking routine (CST) of the DIVA software (Version 7.0, BD Bioscience). 10,000 cells were acquired for each sample and analysis were carried out using FlowJo 10.1.

Instrument	LSRII, BD Biosciences
Software	DIVA software (Version 7.0, BD Bioscience) was used for collection of data<br>FlowJo 10.1. was used for analysis of data.
Cell population abundance	Resting human PBTs were purified via negative-magnetic bead selection with the Pan T-cell isolation kit according to manufacturer`s instructions. Upon purification, cells are stained for CD3 antigen. Purity is determined based on checking the percentage of CD3 positive population.
Gating strategy	Cells were initially gated based on FSC and SSC parameters. Thereafter, singlets were gated by the use of FSC A vs FSC-H parameters. The doublets or other multiplets were gated out. In the following step, Annexin V and 7-AAD positive populations were gated. Autofluorescence (unstained) was used to set the positive populations.

☐ Tick this box to confirm that a figure exemplifying the gating strategy is provided in the Supplementary Information.
